# Optimizing the Management and Outcomes of Failed Back Surgery Syndrome: A Consensus Statement on Definition and Outlines for Patient Assessment

**DOI:** 10.1155/2019/3126464

**Published:** 2019-02-18

**Authors:** Philippe Rigoard, Kliment Gatzinsky, Jean-Philippe Deneuville, Wim Duyvendak, Nicolas Naiditch, Jean-Pierre Van Buyten, Sam Eldabe

**Affiliations:** ^1^Spine & Neuromodulation Functional Unit, Poitiers University Hospital, Poitiers, France; ^2^Institut Pprime UPR 3346, CNRS, ISAE-ENSMA, University of Poitiers, Poitiers, France; ^3^PRISMATICS Lab (Predictive Research in Spine/Neuromodulation Management and Thoracic Innovation/Cardiac Surgery), Poitiers University Hospital, Poitiers, France; ^4^Department of Neurosurgery, Sahlgrenska University Hospital, Gothenburg, Sweden; ^5^Department of Neurosurgery, Jessa Hospital, Hasselt, Belgium; ^6^Department of Anesthesia and Pain Management, Hospital AZ Nikolaas, Sint-Niklaas, Belgium; ^7^Department of Pain and Anaesthesia, The James Cook University Hospital, Middlesbrough, UK

## Abstract

Failed back surgery syndrome (FBSS) is a controversial term for identifying patients affected by new, recurrent, or persistent pain in the low back and/or legs following spinal surgery. The lack of a comprehensive standardized care pathway compromises the appropriate management of FBSS patients, which is associated with a heavy financial burden. An international panel of spine surgeons, neurosurgeons, and pain specialists with a particular interest in FBSS established the chronic back and leg pain (CBLP) network with the aim of addressing the challenges and barriers in the clinical management of FBSS patients by building a common transdisciplinary vision. Based on literature reviews, additional input from clinical expertise of multiple professional disciplines, and consensus among its members, the network attempted to provide recommendations on the management of patients with FBSS utilizing a multidisciplinary team (MDT) approach. The presentation of this work has been divided in two separate parts to enhance its clarity. This first paper, in favour of selecting appropriate validated tools to improve the FBSS patient assessment, focuses on FBSS taxonomy and its clinical implications for evaluation. Concise recommendations for assessment, treatment, and outcome evaluation using a MDT approach would be an important resource for specialists and nonspecialist clinicians who manage patients with FBSS, to improve decision-making, reduce variation in practice, and optimize treatment outcomes in this difficult-to-treat population.

## 1. Introduction

Failed back surgery syndrome (FBSS) is a subcategory of a broader group of pain conditions referred to as chronic back and leg pain (CBLP) [1]. The majority of published definitions for FBSS include new, recurrent, or persistent pain in the back and/or legs following spinal surgery [2–8], with an incidence estimated to be around 20% in the most recent publications on this topic [9, 10]. This is a complex condition with a complicated pathophysiology comprising various aetiologies and pain characteristics that negatively impact function, behaviour, and mental and social well-being [1, 5, 11, 12].

Use of the term FBSS has provoked decades of controversy because of a lack of consensus for a single definition and the inherently restrictive and confusing meaning of this acronym, implying unsuccessful treatment with connotations of blame aimed towards the surgery [12]. A diverse nomenclature has consequently been developed over the last three decades. “Postlaminectomy syndrome” [2, 13], “rebound radicular syndrome” [14], “postoperative persistent pain syndrome” [5], and “chronic postsurgical pain” [15] have been proposed as replacements for FBSS.

The complexity of FBSS suggests that a multidisciplinary strategy to patient assessment, treatment, and therapy evaluation is important for optimization of outcomes [16–19], but no consensus has been clearly defined yet. An international panel of clinicians with a special interest in FBSS established the chronic back and leg pain (CBLP) network and set a list of precise objectives to bridge this gap. The purpose of this paper is to (i) delineate a clear definition of FBSS and specify the criteria for appropriate diagnosis and (ii) suggest recommended treatment evaluation tools to validate and standardize a care pathway for this patient group.

## 2. Materials and Methods

### 2.1. The Chronic Back and Leg Pain Network Constitution and Goals

To address the challenges and barriers in the management of patients diagnosed with FBSS, an international panel of clinical specialists with a specific interest in FBSS established the CBLP network in 2012.

The main goals of the network were to provide consensus on (1) a definition of FBSS, (2) recommendations for validated tools to improve FBSS patient assessment and evaluation of treatment outcomes, and (3) a proposal for a standardized care pathway to support clinicians in their decision-making on how to manage patients with FBSS based on a MDT approach.

Since FBSS remains a standardized subjective term assigned by indexers for both MeSH (MEDLINE/PubMed) and EMTREE (Embase) and since the proposition of a replacement term was not an objective of this paper, the CBLP network focused specifically on the definition of FBSS and patient evaluation and treatment.

### 2.2. Methodology

**Figure 1:**
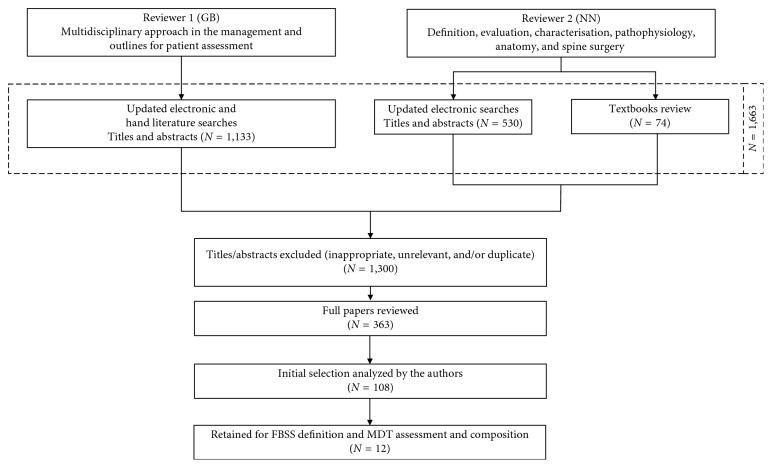
Schematic diagram of steps in the literatures searches: FBSS definition and evaluation. The electronic and hand literature searches yielded 1663 titles and 74 textbooks. Following a review of full-text versions of the 363 residual publications, after discarding duplicates and initial exclusion of 1300 titles/abstracts, 108 papers were finally selected, and 12 were retained for FBSS definition and MDT assessment and composition (Supplementary Material 1).

In order to achieve the set goals, the following methodology was used:

- Participants in the CBLP panel were included based on their extensive clinical experience in managing FBSS patients with a focus on representation from the three specialties that are most involved in the treatment of this patient population: spine surgeons, neurosurgeons, and pain specialists, including anesthesiologists. Invitations were sent to potential participants and accepted prior to formal engagement. Formal face-to-face meetings were held annually between 2012 and 2016 with subsequent teleconferences for an additional feedback prior to drafting the manuscript. The meetings were chaired by a trained facilitator to help the consensus process.
- Literature searches in PubMed, MEDLINE, LILACS, Embase, and the National Guideline Clearing House were conducted by two separate reviewers: one independent reviewer (GB) and one reviewer on the behalf of the clinical group (NN), on a regular basis up to September 2018, without any restrictions regarding language or year of publication. The search strategy was developed in order to maximise sensitivity of article identification, using controlled vocabulary and title/abstract words combining variations of “Failed back surgery syndrome,” “Back pain,” “Chronic leg pain” with “Multidisciplinary” OR “Team,” “Clinical pathway” OR “Practice guideline” OR “Algorithm” OR “Guideline” OR “Protocol,” detailed here after. The independent reviewer (GB) performed a comprehensive electronic search of peer-reviewed full-text papers published between February 2, 2005, and February 21, 2018, in PubMed, MedLine, Embase, and the National Guideline Clearing House. Key words and terms pertaining to the condition (i.e., “failed back surgery syndrome,” “low back pain,” and “leg pain”) were cross referenced with terms pertaining to reports presenting recommendations for MDT involvement in management (i.e., “interdisciplinary communication,” “multidisciplinary” OR “multidisciplinary team” OR “multidisciplinary care” OR “patient referral”) in relevant combinations. Handsearching of reference lists of identified reports and relevant review articles were also carried out. For the group reviewer (NN), the search strategy varied according to the database as follows:
  - *Medline* (“Failed back surgery syndrome” OR “Chronic Back pain” OR “Chronic leg pain”) AND (“Definition” OR “Characterisation” OR “Characterization” OR “Evaluation”)
  - *LILACS* (“Failed back surgery syndrome” OR “Back pain”) AND (“Definition”)
- All references retrieved from databases were exported to Zotero where duplicates were discarded using the “find duplicates” tool.
- In addition, book chapters dealing with “FBSS,” “postoperative low back pain,” and the same controlled vocabulary used previously were initially identified from a systematic review of the electronic literature and of all pathophysiology, anatomy, and physiology textbooks available in the following medical libraries:
  - Paris Medical Library (Université Descartes, Paris 5, rue de l'Ecole de Médecine, 75006 Paris, Fr)
  - Paris Anatomy Library (Anatomy Laboratory, Université des Saints-Pères, Paris 6e, Fr)
  - Poitiers Anatomy Library (Department of Morphology, Poitiers Medical College, rue de la milètrie, 86000 Poitiers, Fr)
  - UIC Library of Health Sciences (University of Illinois at Chicago, 1912 Polk St., Chicago, US)
  - Dorsch Neuroscience Library (Institute of Neurology and Neuropsychiatry, 712 S Wood St., Chicago, US)
- The two literature searches were pooled and converged into one final diagram. The final literature review was conducted to ensure that participants had access to the same body of evidence during the panel discussions. The methodology is summarized in Figure 1.
- Additional input was provided by relevant clinical specialists (psychologist, psychiatrist, physiotherapist, and rehabilitation physician) involved in the multidisciplinary evaluation and treatment of patients with FBSS.
- Consensus was defined as full agreement among the members of the CBLP network on the set goals which was achieved by face-to-face meetings with facilitated round table discussions focusing on the outcome of the literature overview, each member's personal experience, and input from additional clinical specialists. The consensus process did not include any ranking questionnaires based on the Delphi method since the number of participants was considered to be too small and the purpose of the discussions was not to measure consensus based on specific statements but to develop full consensus (resolve disagreement) on the set tasks [20].

## 3. Results

### 3.1. Definition of FBSS

The CBLP network's proposed definition of FBSS is based on the prediction that no further spine surgery is indicated after an appropriate somatic, radiological, and psychosocial assessment [21]. The key elements for definition of FBSS can be summarized in 4 aspects:

1. Back and/or leg pain that persists for at least six months following the most recent spinal surgery
2. The patient has undergone a thorough clinical and radiological assessment
3. There is no clear surgical target on clinical examination and imaging that is concordant with presenting symptoms
4. There is interdisciplinary agreement that additional surgical intervention (decompression and/or fusion) is not appropriate

It is important to ensure that the temporal relationship between the most recent surgery and the presentation of pain is explored adequately so that complications that are known to occur within the first six months of surgery (e.g., hardware failure, recurrent herniation, and infection, including discitis and abscess) can be identified and taken care of promptly [5]. As a consequence, our definition of FBSS is found on the generally accepted definition of chronic pain in the context of surgery as “pain that persists 6 months after an injury and beyond the usual course of an acute disease or a reasonable time for a comparable injury to heal” [22].

### 3.2. Multidisciplinary Approach in the Management of FBSS

Out of 12 pain-related guidelines or health technology assessments that were identified in the literature overview, nine recommended the practice of involving a MDT as the standard-of-care [12, 23–30]. Importantly, a previous meta-analysis of 41 randomized controlled trials (RCTs) (*N* = 6,858) yielded by a systematic literature review up to March 2014 revealed that a MDT approach was significantly more effective than usual care in reducing pain and disability in patients with chronic low back pain [31].

In a general statement about pain management, the International Association for the Study of Pain (IASP) recommended that, “clinicians who assess and treat patients in a pain center should include physicians, nurses, mental health professionals (e.g., clinical psychologist and psychiatrist), and physical therapists” [23]. However, many patients do not have access to a pain center, making access to a MDT challenging. To date, recommendations for the composition of a FBSS-specific MDT provided by governments and experts alike suggest the involvement of a neurologist, a rheumatologist, a pain physician, a spine surgeon, a neurospine surgeon, a functional neurosurgeon, a rehabilitation physician, a radiologist, a physiatrist, a pain nurse, and a psychologist/psychiatrist [12, 17, 24].

Based on the literature review (Table 1), additional specialist input and consensus among its members, the CBLP network recommends that a FBSS-oriented MDT should include five types of health professionals, reflecting the continuum of the FBSS patient care pathway:

- One or several “pain physician(s)” (i.e., anaesthetist, rheumatologist, and neurologist) representing the cornerstone of professional interactions for the assessment and treatment of pain, focusing on optimizing medical and interventional management within the FBSS care pathway
- One or several rehabilitation physicians, physiotherapist(s), and/or physiatrist(s) to optimize physical examination and review potential rehabilitation strategies
- One or several psychologist(s) and/or psychiatrist(s) to focus on psychosocial aspects and supply ongoing psychological evaluation and support [35, 36]
- One or several “spine surgeon(s)” (i.e., neurosurgeon and orthopaedic surgeon) supported by a radiologist to provide an ultimate spine expertise, making sure that no further surgery is required helping to characterize the pathophysiology of pain generators
- One or several member(s) of a “neuromodulation team” (i.e., implanter/anaesthesiologist/neurosurgeon and pain/neuromodulation nurse) to evaluate the eligibility for neurostimulation/intrathecal drug delivery (IDD) therapies in the context of a refractory patient

### 3.3. Initial MDT Evaluation

The initial stage in the proposed care pathway by the CBLP network is based on MDT evaluation to confirm the FBSS diagnosis. With a specific reference to our definition of FBSS, the clinical work-up of a patient should include the following: (a) history to confirm the occurrence of prior spinal surgeries; (b) precise physical examination; (c) carefully chosen diagnostic tools to assess pain severity and functional incapacity; (d) psychosocial evaluation; (e) appropriate diagnostic radiological assessment.

#### 3.3.1. Pain History

The pain history must be carefully reviewed and pay specific attention to the following items:

- New pain, persistent pain, and/or recurrent pain? (if yes, free interval needs to be quantified)
- Circumstances, positions, and movements that improve or exacerbate the symptoms [17]
- Any clinical sign that might be suspicious of underlying infection, neoplasm, and fracture, called “red flag” by the COST B13 group in available European guidelines [37]

#### 3.3.2. Physical Examination

It remains difficult to transpose a “mechanistic” concept based on pathophysiological evaluation of pain into daily practice. Nevertheless, this approach plays a critical role to better understand FBSS management. Several authors have tried to identify, from all of the findings of spine examination, those clinical signs that are predictive of a particular type of lesion (facet joint and discogenic or muscle pain, for example), taking into account pain typology (mechanical/neuropathic) [5]. This type of pathophysiological segmentation might be used to predict the quality and the magnitude of response to the various treatments proposed (classical response to opioids in nociceptive pain and efficacy of antiepileptic drugs or neurostimulation in neuropathic pain, for example). An emphasis on the spatial dimension concerning the topographic distribution of the pain should be incorporated into the concept of pathophysiological pain characterization. Thus, chronic leg pain persisting despite a surgical decompression might be frequently associated to a neuropathic pain component, while the pain characterization becomes much more complex when trying to interpret axial pain, where biomechanical and neuropathic components appear to be inseparable or difficult to isolate [1]. Even though promoting strict dichotomy between the leg pain and the back pain components might be artificial, this clinical strategy can nevertheless guide the physician in the clinical evaluation.

*(1) The Leg Pain Component*. The first step of leg pain clinical examination aims to confirm a radicular lesion mainly indicative of neuropathic pain and to eliminate pain due to another cause. The presence of sensory dysfunction (hypoesthesia, anesthesia, or allodynia) in the painful territory and/or motor deficit is suggestive of a nerve lesion and thus a neuropathic pain. Several diagnostic tools have been validated for neuropathic pain detection: DN4 (Douleur Neuropathique in 4 questions), LANSS (Leeds assessment of neuropathic symptoms and signs), or S-LANSS (simplified version) [38, 39]. The DN4 questionnaire, published in 2005, is easy to use in daily practice. It is composed of four questions comprising a total of seven items scored during the clinical interview and three items based on physical examination [38]. A score greater than or equal to 4/10 is pathognomonic of neuropathic pain with a sensitivity of 82.9% and a specificity of 89.9%. Shamji and Shcharinsky [40] recently stated that a positive DN4 questionnaire is a powerful predictor of spine (re)surgery failure. The presence of mechanical signs of disc-nerve root conflict (impulse pain, Lasègue's sign) suggests a physical conflict at the level of the disc (disc herniation recurrence and residual conflicting elements) or at the level of the foramen (foraminal stenosis and segmental spine instability) [17] and requires a specific spine expertise. Hip, knee, and sacroiliac joints examination are important to avoid the classical diagnostic traps associated with pain of the anterior surface of the thigh or trunked S1 sciatica. A vascular examination has to be performed in case of intermittent claudication on walking. A careful palpation of the sciatic trunk eliminates a neurinoma and a piriformis syndrome at the gluteal region.

*(2) The Back Pain Component*. In contrast to leg pain assessment, and before considering any potential neuropathic aspect of the back pain (first described by Attal et al. in 2011) [41], clinical investigation of the back pain component in FBSS patients should be based on meticulous dissection of all potential mechanical triggers that could be a source of the nociceptive pain characteristics [11]. Examination of the spine aims to assess the global posture and stability of the spine in the different planes. Meticulous palpation aims to identify a possible vertebral or paravertebral pain trigger point. The main focus of the back assessment is guided by a somatic diagnostic process using validated diagnostic rules [42]. The key potential spinal pain generators, which need to be carefully reviewed, are myofascial syndrome, the facets, and the disc complex.

- Muscle pain: the muscle trophicity may be compromised by chronic degeneration related to the initial spine pathology, by physical hypoactivity, and by repeated surgery, leading to potential and progressive instability of the vertebral column [43]. A vicious circle can occur when biomechanical overloading (created by disease progression and/or hardware failure) causes displacements of the spine and induces new tensions. Results show increased impulse activity of muscle and tendon nociceptors establishing the neurophysiological basis of postoperative muscle pain.
- Facet joint pain and spinal instability: spine surgery induces major changes in the biomechanical loads on all surrounding structures [44]: muscles, ligaments, discs, facet joints, fat tissues, and fascia. This mechanical loading redistribution occurs particularly in adjacent spinal segments, around the anterior or posterior instrumentation in case of stabilization [11]. Foraminal residual stenosis can be responsible for persistent nerve entrapment, as mentioned above: anteriorly from vertebral spurs and posteriorly from facet arthritic changes. As a consequence, patient's symptoms can be linked to facet compensation syndrome [45, 46], but isolating facets as the specific source of pain is difficult in FBSS patients.
- Discogenic pain: it is predominantly described as a deep midline low back pain with a bilateral metameric irradiation. Mechanical and positional in nature, the pain is generally worse when upright rather than supine [47]. It is thought that this type of pain is generated by the visceral afferents that innervate the intervertebral disc, known as “the sinu-vertebral nerve” [48].

#### 3.3.3. Validated Tools for Assessment of Pain and Functional Parameters

In line with the adoption of a uniform MDT approach to the management of FBSS, it is important to utilize recommended validated tools to evaluate treatment outcomes in patients with pain disorders in compliance with IMMPACT recommendations [49, 50]. The aims of a standardized assessment in this context are to facilitate longitudinal patient evaluation, to increase the efficiency, clarity, and quality of patient information during the referral process and to improve dialogue between centers and disciplines in order to optimize treatment decisions. The aims of a common approach to treatment evaluation are therefore relevant to patients, clinicians, payers, and policymakers [51].

The CBLP network recognizes that treatment evaluation should involve previously issued quality standards and guidelines for documentation of outcomes including the patient's subjective assessment of pain severity, function, and health-related quality of life (HRQoL) [49, 52]. Having considered the plethora of existing tools used to evaluate these constructs from the patient's perspective, clinical experience guided the CBLP network to recommend a choice of standardized and validated instruments (Table 2).

For pain severity scales, there is a choice of the visual analogue scale (VAS) or the numeric pain rating scale (NPRS) [53–55]. To assess pain-specific disability/function, either the Oswestry Disability Index (ODI) [56] or the Roland Morris Disability Questionnaire (RMDQ) [57] is recommended. To measure generic HRQoL, either the EuroQol with five dimensions (EQ-5D) [58] or the short form 12/36 (SF-12/SF-36) [59, 60] is recommended. The shorter generic HRQoL instruments, the SF-12 and the EQ-5D, minimize patient burden. It is also recommended that clinicians continuously monitor and evaluate a patient's medication intake.

#### 3.3.4. Psychosocial Assessment

It is well established that psychological factors affect pain perception [61] and clinical outcomes [48, 62–66]. There is no accepted gold standard approach for the psychological screening of FBSS patients. Optional questionnaires can be used to assist the psychological assessment. The most recognized questionnaire in this context is the Minnesota Multiphasic Personality Inventory 2 Restructured (MMPI-2-RF) [67–70]. The Hospital Anxiety and Depression Scale (HADS) allows detection of various states of depression (HADS-D) and anxiety (HADS-A) [71–73], and the Fear Avoidance Beliefs Questionnaire Work and Activity (FABQ) measures patient's fear of pain [74].

Coping strategies may play an important role by determining how patients cooperate with chronic symptoms and with pain management [75, 76]. The Coping Strategies Questionnaire (CSQ) is intended to measure six cognitive and two behavioural coping strategies. Active coping strategies are linked to positive effect, better psychological adjustment, and decreased depression, while passive strategies are linked to poorer outcomes such as depression and increased level of pain [65, 77, 78]. An important subscale of CSQ is catastrophizing. Based on the CSQ, Sullivan and colleagues [79] developed the Pain Catastrophizing Scale (PCS). The PCS is widely used, and elevated scores have been associated with poor treatment outcomes.

The optimization of chronic pain management requires consideration of the social factors that may contribute to people's physical and mental health [80–83]. Factors such as gender, level of education, and working status play a substantial role in pain perception and affect patient compliance and their pain management [65, 84–88]. Medical professionals trained to solve a physical/somatic problem, where there is a potential biopsychosocial comorbidity may fail to anticipate and manage the vicious circle of social exclusion. These arguments support the need for including social assessment in a MDT approach, as it affects clinical outcome in chronic pain management [89].

A proposal for a minimal psychosocial assessment toolbox is presented in Table 3.

#### 3.3.5. Radiological Assessment

In addition to orthopaedic, neurologic, functional, and psychosocial evaluation, spine imaging is essential in order to exclude new indications for reoperation: recurrent disc herniation (MRI), spine instability (CT, MRI, and bending X-ray), spine imbalance (full-standing lateral and anteroposterior X-ray), EOS® images while the patient is standing (bending X-ray), or nonunion of spinal fusion (plain X-ray, CT, scintigraphy, and positron emission tomography (PET) scan), new-onset stenosis (MRI and CT), and abscess (MRI and CT) [17, 90]. Conditions like discitis, low-grade infections, arachnoiditis, or scar tissue which usually do not require reoperation have also to be identified before being managed conservatively [18].

**Figure 2:**
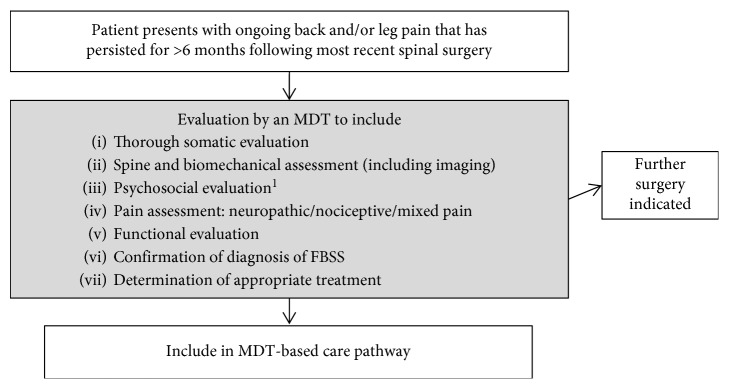
The initial stage of the proposed standardized multidisciplinary team failed back surgery syndrome care pathway, as recommended by the Chronic Back and Leg Pain Network. Clinical evaluation and confirmation of diagnosis. FBSS, failed back surgery syndrome; MDT, multidisciplinary team. ^1^Best practice is for the psychosocial evaluation to be performed by a psychologist or psychiatrist with specific experience in the field of pain. Assessment should include the relevant tests and questionnaires that are able to identify patients with major psychological or psychiatric contraindications (Table 3).

Following a robust MDT clinical evaluation to define and diagnose a patient with FBSS (Figure 2), a stratification and hierarchization of the various therapeutic options can be constructed through a level approach to optimize management and outcomes. A patient who either does not present with FBSS or presents with FBSS and a significant psychosocial comorbidity, determined by psychological assessment carried out by a clinical psychologist or psychiatrist (ideally with experience in the field of pain), should be excluded from the pathway and referred to the appropriate discipline(s).

## 4. Discussion

Use of the term FBSS has provoked decades of controversy because of a lack of consensus of a definition and the inherently restrictive and confusing meaning of this acronym. The term hides the challenges associated with selecting appropriate treatment for this patient population due to insufficient identification of the underlying mechanism of pain [4, 5]. In addition, cognitive, affective, and behavioural features of pain are often explanations of the disability as much as or more than abnormal sensory-related pain [4].

In response to the limitations of current practice in managing FBSS patients, an international panel of spine surgeons, neurosurgeons, and pain specialists with a special interest in FBSS established the CBLP network. Based on the broad personal experience of each member of the panel, through literature reviews, additional input from clinical expertise of multiple professional disciplines and consensus among its members, the CBLP network's primary intention is to provide recommendations on how to optimize the management and outcomes of FBSS.

In this paper, we focus on the key elements for defining FBSS and outline how to clinically confirm this diagnosis. The CBLP network's definition of FBSS is found on the prediction that no further spine surgery is indicated after adequate somatic, psychosocial, and radiological assessment have been executed. Management begins with a systematic evaluation of common FBSS aetiologies. Appropriate understanding and identification of the abnormalities most commonly associated with FBSS after a meticulous clinical evaluation is required for adequate caretaking of this often hard-to-treat condition [1, 4]. A growing body of data suggests that the adoption of a multidisciplinary approach is significantly more effective than usual care of patients with chronic low back pain [25, 31, 91, 92]. A MDT-based approach has the potential to improve decision-making, reduce variation in practice, and optimize treatment outcomes. Clinicians should refine pain topographical and topological pain characterization to ensure that clinical evaluation becomes the guiding principle for multidisciplinary assessment. A patient who either does not present with FBSS or presents with FBSS and significant psychosocial comorbidities should be excluded from further inclusion in FBSS care pathways or algorithms and be referred to the appropriate discipline(s).

For many aspects of medical practice, there is a lack of high-quality evidence and a plethora of contradictory information which makes decision-making difficult when trying to optimize treatment outcomes and provide concise recommendations for assessment, treatment, and outcome evaluation. When dealing with a lack of, or conflicting, scientific evidence, consensus statements are seen as a useful tool to establish expert agreement, to define the boundaries of acceptable practice and obtain opinions from different countries and healthcare systems [20, 93]. Due to the paucity of evidence-based guidelines in the management of FBSS, the CBLP network chose to adhere to a consensus-based approach to achieve the set goals to define FBSS, design outlines for appropriate patient evaluation, and propose a treatment pathway. One limitation of the chosen method to achieve consensus in the present study is that it was based on round table discussions without involvement of ranking procedures that are used in the two most commonly utilized methods to reach consensus: the Delphi process and the nominal group technique [20]. Although not optimal, we consider the method we adopted to be appropriate based on the complexity of the set tasks which do not fit easily within an evidence-based treatment paradigm. Since personal contact with facilitated discussions among the network members was considered desirable, the Delphi method, the reliability of which rests on anonymity and increases with the size of the group, was not used [94].

## 5. Conclusions

The complexity of FBSS suggests that a multidisciplinary strategy is most appropriate for patient assessment with the goal to optimize outcomes. This paper focuses on redefining FBSS taxonomy and clinical evaluation in order to improve patient assessment before adequate treatment options can be chosen. It is important for physicians and other healthcare professionals involved in the management of patients with FBSS to expand their knowledge of underlying aetiologies and use of appropriate diagnostic tools to adequately evaluate this difficult-to-treat group affected with chronic pain. In a second paper, a stratification and hierarchization of the various therapeutic options is constructed through a 4 level-approach with a proposal for a standardized treatment pathway for FBSS. The utilization of a MDT approach is emphasized to ensure that care is provided in a uniform manner for optimizing management and ultimately patient outcomes.

## Figures and Tables

**Table 1:** Output of the review of publications with recommendations for specialties to be included in the multidisciplinary team management of failed back surgery syndrome.

Manuscript identification	Design	Management: specialities
Amirdelfan et al. [32]	Evidence-based approach—literature review (guideline)	Physiotherapist
		Psychologist
		Interventional pain physician
		Neurosurgeon trained to implant neuromodulation options

Al Kaisy et al. [12]	Expert consensus (algorithm)	Neurologist
		Rheumatologist
		Oncologist
		Pain physician
		Psychiatrist
		Rehabilitation physician
		Functional neurosurgeon
		Physiotherapist/physiatrist
		Psychologist
		Spine surgeon

Baber and Erdek [18]	Discussion of the literature	Physical therapist
		Psychologist
		Pain specialist
		Spine surgeon
		Primary care provider

Chan and Peng [8]	Expert consensus	Psychologist
		Occupational therapist
		Spine surgeon
		Physiotherapy

Desai et al. [33]	Literature review	Psychologist
		Physiotherapist
		Medical (not defined further)
Ganty and Sharma [34]	Expert consensus (algorithm)	Pain physician
		Psychologist
		Physiotherapist
		Neuromodulation nurse
		Spine surgery
		General physician

Hussain and Erdek [16]	Expert consensus	Physiotherapist
		Psychologist

Rigoard et al. [5]	Expert consensus	Pain physician
		Anesthesiologist
		Surgeon

**Table 2:** Validated questionnaires recommended for completion by patients to assess pain severity, function, and health-related quality of life.

(i) Pain scales
Visual analogue scale (VAS): 10 cm leg VAS and back VAS *OR* the numeric pain rating scale (NPRS) [53–55]
(ii) Pain-specific disability/function
Oswestry disability index (ODI) [56] *OR* Roland Morris disability questionnaire (RMDQ) [57]
(iii) Generic health-related quality of life
EuroQol 5 dimensions (EQ-5D) [58]
Short form 12 (SF-12) *OR* short form 36 (SF-36) [59, 60]

**Table 3:** Instruments to assess psychological and social well-being among patients with failed back surgery syndrome (FBSS).

	Psychological assessment	Social assessment
Recommended	Minnesota Multiphasic Personality Inventory 2 Restructured (MMPI-2-RF)	Age, gender, educational level, and working status
	The Hospital Anxiety and Depression Scale (HADS)	Social class
	The Fear Avoidance Beliefs Questionnaire Work and Activity (FABQ)	Financial incomes
	The Coping Strategies Questionnaire (CSQ)	Marital status and social withdrawal
	The Pain Catastrophizing Scale (PCS)	

## Supplementary Materials

Supplementary Materials Supplementary Material 1 (with a reference list) presents a description of the 12 reports yielded by literature searches relating to recommendations for the management of failed back surgery syndrome involving multidisciplinary teams.
