# Supplementary material for: Optimizing the Management and Outcomes of Failed Back Surgery Syndrome: A Consensus Statement on Definition and Outlines for Patient Assessment
Source: Pain Res Manag. 2019 Feb 18;2019:3126464. doi: 10.1155/2019/3126464 (PMC6398030; doi:10.1155/2019/3126464)
Supplement: Supplementary Materials — Supplementary Material 1 (with a reference list) presents a description of the 12 reports yielded by literature searches relating to recommendations for the management of failed back surgery syndrome involving multidisciplinary teams. [file 3126464.f1.docx]

# Supplementary Material

| **Society/ group/ agency (year of publication)** | **Geography** | **Focus of report** | **Recommendations** | |
| --- | --- | --- | --- | --- |
|  |  |  | **Multidisciplinary management** | **Failed back surgery syndrome (FBSS)** |
| American Pain Society (2007) [1] | US | Low back pain | “*For significant functional deficit, consider more intensive multidisciplinary approach or referral.*” | - |
| British Pain Society in consultation with the Society of British Neurological Surgeons (2009) [2] | United Kingdom | Spinal cord stimulation for the management of pain: recommendations for best clinical practice | “*Multidisciplinary pain services should offer a range of evidence-based interventions to patients with persisting pain. It is rarely possible to provide complete pain relief”* | “*There is clinical evidence from randomised controlled trials to support use of SCS in pain from failed back surgical syndrome (FBSS), complex regional pain syndrome (CRPS), neuropathic pain, and ischaemic pain*.” |
| International Association for the Study of Pain – IASP (2009) [3] | International | Pain in general | “*Patient assessment and treatment should be multidisciplinary, involving appropriate specialists as needed, to ensure optimal management of all biomedical and psychological aspects of pain problems.*” | - |
| American Society of Anesthesiologists Task Force (2010) [4] | US | Chronic pain overall | “*Multimodal or Multidisciplinary Interventions:*   - *Multimodal interventions should be part of a treatment strategy for patients with chronic pain.* - *A long-term approach that includes periodic follow-up evaluations should be developed and implemented as part of the overall treatment strategy.* - *When available, multidisciplinary programs may be used.*”   “*The literature indicates that the use of multidisciplinary treatment programs compared with conventional treatment programs is effective in reducing the intensity of pain reported by patients for periods of time ranging from 4 months to 1 yr (Category A2 evidence, supported by multiple RCTs but insufficient number to perform a viable meta-analysis). The literature is insufficient to evaluate comparisons of multimodal therapies with single modality interventions (Category D evidence, insufficient evidence from the literature).*” | “*SCS may be used in the multimodal treatment of persistent radicular pain in patients who have not responded to other therapies.”*  *SCS in combination with physical therapy is superior to physical therapy alone (Category A3 evidence, supported by 1 RCT) and provides more pain relief when compared to reoperation (Category A3 evidence, supported by 1 RCT).*” |
| International expert consensus (2010) [5] | International (from the series: Evidence-based Interventional Pain Medicine According to Clinical Diagnoses”) | Lumbosacral Radicular Pain - FBSS | - | In patients with a therapy-resistant radicular pain in the context of a FBSS, SCS (i.e., not revision surgery) is recommended and should be performed in specialized centers |
| Minister of Public Health and Social Affairs (2012) [6] | Belgium | Severe chronic pain – SCS and intrathecal analgesic delivery pumps | “*The management of severe chronic pain inherently needs a multidisciplinary approach including various medical and para-medical specialties, depending upon the origin of the pain.*” | ”*Neuromodulation can only be considered in selected patients after having completed a full assessment by a truly multidisciplinary team of pain specialists in an experienced and specialised pain centre*” |
| Health Technology Agency (2013) [7] | Ireland | Spinal cord stimulation (SCS) for chronic pain | “*SCS should be delivered, with other therapies, through a multidisciplinary pain management team*” | “*There is good quality evidence that SCS is more effective than continued conservative management or repeat operation in reducing pain at six months and 12 months, for individuals with FBSS*” |
| International Association for the Study of Pain Neuropathic  Pain Special Interest Group - NeuPSIG (2013) [8] | International | Interventional management of neuropathic pain  ‘Interventional ‘defined as invasive procedures involving delivery of drugs into targeted areas, or ablation/modulation of targeted nerves | **-** | “*We did not find studies assessing the efficacy of epidural steroid injection for treating patients with FBSS who have prominent radicular symptoms (‘‘inconclusive’’ recommendation).*”  “*Although adhesiolysis may help some patients with FBSS, we conclude that the evidence of efficacy for NP [neuropathic pain] associated with FBSS is uncertain and give an ‘‘inconclusive’’ recommendation.*”  “*Based on the strength of these trials* [two RCTs]*, an independent systematic review concluded that SCS appears to be more effective than CMM* [conventional medical management] *and reoperation.”* (quality of evidence: moderate; strength of evidence: weak) |
| Expert consensus (2015) [9] | UK, US, France | FBSS | “*Ideally, it could be proposed that the patient would derive optimal benefit from systematic confrontation of these various points of view [pain physicians, orthopaedic spine surgeon, neuro spine surgeon, functional neurosurgeon, physiatrist, psychologist and health economist] in order to propose the best treatment option at a given point in time to achieve the best possible care pathway.* “  “*All these pain-relieving procedures* [pharmacological therapies, epidural steroid injection, radiofrequency denervation, trigger point injections, epidural adhesiolysis, SCS] *should be used in the context of multidisciplinary care with the overall aim of providing the patient with tools to manage their pain with physical and psychological rehabilitation.*” | “*Pharmacological therapies are the most common medical approach to pain relief and consist of both conventional analgesics and anti-neuropathic medications, such as the gabapentinoids and tricyclic antidepressants. These should be used in the context to helping patients manage their pain and are not agents that should be used alone in the long-term as tolerance and side effects are common.*  *Interventional pain therapies, such as epidural steroid injection, radiofrequency denervation, trigger point injections and epidural adhesiolysis are frequently used but the evidence for their efficacy in FBSS is weak*  *Neuromodulation, such as spinal cord stimulation for FBSS has amongst the highest grade of evidence in pain-relieving treatments. The two high quality randomized trials show that this approach is superior to conventional medical management and repeat surgery.*” |
| German National Disease Management Guideline (2015) [10] | Germany | The management of non-specific low back pain | “*In the event of sustained pain (>12 weeks), continue with somatic diagnostics and assess psychosocial influencing factors (optimally on the basis of inter-/multidisciplinary assessments)*.”  “*The long-term care of patients with low back pain should be provided by inter-/multidisciplinary teams*.”  ”*Inter and multidisciplinary assessments should combine at least medical (pharmacotherapy, education), physical (exercise), work-related and behavioural-therapy components.*” | - |
| European Association of Neurologists (2016) [11] | Europe | Neurostimulation therapy for neuropathic pain | - | ”*There is weak recommendation for the use of SCS added to conventional medical management versus conventional medical management in … CLBP … and for the use of SCS as an alternative to reoperation in post-surgical CBLP.*” (low or moderate quality of evidence) |
| Health technology agency (2016) [12] | England and Wales | Low back pain and sciatica in over 16s: assessment and management | The guideline includes recommendations for the assessment and management of low back pain or sciatica in people aged 16 and over outlining physical, psychological, pharmacological and surgical treatments. | - |

**References**

1. Chou R, Qaseem A, Snow V, Casey D, Cross JJ, Shekelle P, Owens D, Clinical Efficacy Assessment Subcommittee of the American College of Physicians, American College of Physicians, Panel APSLBPG. Diagnosis and treatment of low back pain: A joint clinical practice guideline from the American College of Physicians and the American Pain Society. *Ann Intern Med* 2007;**147**(7)**:** 478-91.

2. The British Pain Society (BPS). Spinal cord stimulation for the management of pain: Recommendations for best clinical practice *2009; Available at:* [*https://wwwbritishpainsocietyorg/static/uploads/resources/files/book_scs_main_1pdf*](https://wwwbritishpainsocietyorg/static/uploads/resources/files/book_scs_main_1pdf) *(accessed Mar 7, 2016)*.

3. International Association for the Study of Pain (IASP). Education. Pain treatment services. *2009; Available at:* [*https://wwwiasp-painorg/Education/Contentaspx?ItemNumber=1381*](https://wwwiasp-painorg/Education/Contentaspx?ItemNumber=1381) *(accessed March 9, 2016)*.

4. Practice Guidelines for Chronic Pain Management. An Updated Report by the American Society of Anesthesiologists Task Force on Chronic Pain Management and the American Society of Regional Anesthesia and Pain Medicine*. *Anesth* 2010;**112**(4)**:** 810-33.

5. Van Boxem K, Cheng J, Patijn J, Van Kleef M, Lataster A, Mekhail N, Van Zundert J. 11. Lumbosacral Radicular Pain. *Pain Pract* 2010;**10**(4)**:** 339-58.

6. Belgian Health Care Knowledge Centre (KCE). Neuromodulation for the management of chronic pain: implanted spinal cord stimulators and intrathecal analgesic delivery pumps (189C). *2012; Available at:* [*https://kcefgovbe/sites/default/files/atoms/files/KCE_189C_neuromodulation_chronic_pain_0pdf*](https://kcefgovbe/sites/default/files/atoms/files/KCE_189C_neuromodulation_chronic_pain_0pdf) *(accessed March 8, 2016)*.

7. HIQA (Health Information and Quality Authority). Spinal Cord Stimulation for chronic pain. *Health Technology Assessment of Scheduled Procedures*. Dublin. 2013. *Available a:t* <https://www.hiqa.ie/sites/default/files/2017-01/Spinal_cord_stimulation_2013.pdf> *(accessed Mar 8, 2016)*.

8. Dworkin RH, O’Connor AB, Kent J, Mackey SC, Raja SN, Stacey BR, Levy RM, Backonja M, Baron R, Harke H, Loeser JD, Treede R-D, Turk DC, Wells CD. Interventional management of neuropathic pain: NeuPSIG recommendations. *Pain* 2013;**154**(11)**:** 2249-61.

9. Al Kaisy A, Pang D, Desai M, Pries P, North R, Taylor R, Mc Cracken L, Rigoard P. Failed back surgery syndrome: Who has failed? *Neurochirurgie* 2015;**61**(Suppl 1)**:** S6-S14.

10. Bundesärztekammer - German Medical Association (BÄK) KB-NAoSHIPK, Arbeitsgemeinschaft der Wissenschaftlichen Medizinischen Fachgesellschaften - Association of Scientific Medical Societies (AWMF),. National Disease Management Guideline 'Low back pain' - Short version. *2015; Available at:* [*http://wwwleitliniende/mdb/downloads/nvl/kreuzschmerz/archiv/kreuzschmerz-1aufl-vers5-shortpdf*](http://wwwleitliniende/mdb/downloads/nvl/kreuzschmerz/archiv/kreuzschmerz-1aufl-vers5-shortpdf) *(accessed Mar 7, 2016)*.

11. Cruccu G, Garcia-Larrea L, Hansson P, Keindl M, Lefaucheur JP, Paulus W, Taylor R, Tronnier V, Truini A, Attal N. EAN guidelines on central neurostimulation therapy in chronic pain conditions. *Eur J Neurol* 2016;**23**(10)**:** 1489-99.

12. National Institute for Health and Care Excellence (NICE). Low back pain and sciatica in over 16s: assessment and management. *2016; Available at:* [*https://wwwniceorguk/guidance/ng59/resources/low-back-pain-and-sciatica-in-over-16s-assessment-and-management-pdf-1837521693637*](https://wwwniceorguk/guidance/ng59/resources/low-back-pain-and-sciatica-in-over-16s-assessment-and-management-pdf-1837521693637) *(accessed March 7, 2017)* (NG59).
